# LoRaWAN and Urban Waste Management—A Trial

**DOI:** 10.3390/s21062142

**Published:** 2021-03-18

**Authors:** Nuno Cruz, Nuno Cota, João Tremoceiro

**Affiliations:** 1 FIT—Future Internet Technologies, ISEL—Instituto Superior de Engenharia de Lisboa, IPL—Instituto Politécnico de Lisboa, 1959-007 Lisboa, Portugal; ncota@deetc.isel.ipl.pt; 2 LASIGE, Faculdade de Ciências, Universidade de Lisboa, 1749-016 Lisboa, Portugal; 3 Câmara Municipal de Lisboa, 1600-036 Lisboa, Portugal; joao.tremoceiro@cm-lisboa.pt

**Keywords:** LoRa, LoRaWAN, trial, waste management, smart city, Internet of Things

## Abstract

The city of Lisbon, as any other capital of a European country, has a large number of issues regarding managing waste and recycling containers spread throughout the city. This document presents the results of a study promoted by the Lisbon City Council for trialing LPWAN (Low-Power Wide-Area Network) technology for the waste management vertical under the Lisbon Smart City initiative. Current waste management is done using GSM (Global System for Mobile communications) sensors, and the municipality aims to use LPWAN in order to improve range and reduce costs and provisioning times when changing the communications provider. After an initial study, LoRa (Long Range) and LoRAWAN (LoRa Wide Area Network) as its network counterpart, were selected as the LPWAN technology for trials considering several use cases, exploring multiple distances, types of recycling waste containers, placements (underground or surface) and kinds of commercially available waste level measurement LoRa sensors. The results showed that the underground waste containers proved to be, as expected, the most difficult to operate correctly, where the container itself imposed attenuation levels of 26 dB on the LoRa link budget. The successful results were used to promote the deployment of a city-wide LoRa network, available to all the departments inside the Lisbon City Council. Considering the network capacity, the municipality also decided to make the network freely available to citizens.

## 1. Introduction

Lisbon, as many other large cities, manages its waste by employing a number of systems associated to the smart city paradigm, such as monitoring waste levels in the containers and optimizing waste truck routes for each of the recycling waste types (common, organic, paper, plastic, and glass). The current system is based on legacy cellular technology, that as a legacy technology, poses a number of technical challenges and costs to the Lisbon City Council.

Within the scope of the Lisbon Smart City project, underway at the Lisbon City Council (CML), it is intended to provide the city of Lisbon with an LPWAN (Low-Power Wide-Area Network) network, which will cover the entire city by making available a platform for low-speed and low-power communications with the set of sensors integrated in the municipality, following the principles of the Internet of Things (IoT). This network will allow the municipality to integrate a set of vertical applications, supported by a common communications network and platform, which will allow greater cost-effectiveness among different departments (and corresponding verticals) as opposed to the current data-as-a-service model, where each provider included a different network in their tender proposal.

In order to support the smart city verticals, different LPWAN technologies that could be adopted by the municipality are currently available on the market. Among the possible alternatives [1], the following stand out:

- LoRaWAN (Low Range Wide Area Network)—the complementary network layer to the LoRa technology, which, by itself, only specifies the physical layer of the communications stack. This technology will be described in Section 2;
- Sigfox—an alternative network with the same aim, based on a closed business model, in which the network is always supported by an operator, also called Sigfox. This is the key difference to other technologies, there is only one operator;
- NB-IoT (Narrow Band IoT)—technology supported by public mobile communications networks, resulting from an evolution of the widely deployed LTE (Long-Term Evolution) technology. NB-IoT is the most recent IoT technology, its operating model is based on the conventional models of public mobile operators, based on a subscription.

One of the smart city vertical applications in the municipality, with a project already underway, consists of sensing the city’s waste containers. This project is supported by a set of sensors installed in the waste containers, which periodically report information on the filling level, this information is then integrated into a central management platform for this type of application. Communication with the installed set of sensors is currently supported through modems that use public mobile communications networks (based on GPRS—General Packet Radio Service) to transmit the container waste level measurement data.

### 1.1. Purpose

Taking into account the knowledge obtained from the existing sensor network, CML determined that strategically it should define a Municipal plan for building a common LPWAN to address all smart city verticals. Within this scope, CML requested ISEL (one of the oldest engineering higher education institutes in Portugal) to study the applicability of the LoRa technology in order to support this network. As a use case, the objective was to determine if LoRa was able to address the most demanding scenarios. The use case was then set to be the transmission of information for monitoring the filling level of waste containers used in recycling throughout the city, including the demanding underground waste containers, where GSM/GPRS was not able to be used without introducing physical adaptations on the containers, that consisted of drilling holes to allow an antenna to be installed on the outside of the container. Using GSM also introduces other disadvantages regarding costs and a large management overhead whenever a new mobile operator wins the public tender. A new public tender is required by Portuguese law after at most three years, which is the maximum lifetime of public procurements. One could assume that GSM has the advantages of not requiring CML to manage the network, however, LoRa can also be installed on the city as a service, provided by a tenderer. Reprovisioning of LoRa devices is easier than GSM for CML, if the LoRa network provider changes after a new tender, no SIM (Subscriber Identity Module) are required to be exchanged at each device. The objective of this document is then to present the results of the tests carried out that proved the technical feasibility of applying LoRa in the typical waste management smart city vertical application, in order to provide grounds for a city-wide LoRa network deployment. The success of these trials was used to determine the deployment of the city-wide network (See: https://lisboainteligente.cm-lisboa.pt/lxi-noticias/lora-a-nova-rede-de-lisboa/ (in Portuguese)). In spite of the main objective of this network being the support of the municipally verticals, the network will also be freely open for citizens to use their own sensors and applications.

### 1.2. Organization of the Document

The document is organized in five sections, starting with an introduction to LoRa technology in Section 2, followed by related work in Section 3. Section 4 presents the performed trials, including the sensors used and use cases for the definition of the different test scenarios. In Section 5 the main evaluation results are presented and finally in Section 6 the conclusions are presented.

## 2. LoRa Technology—Long Range

LoRa technology is a long-range, low-power wireless transmission technology that defines the physical layer of communication between devices, operating in an unlicensed band (in Europe it operates in the 863–870 MHz band). This is the underlying technology of the LoRaWAN protocol [2], which specifies the upper layers of communication.

The main characteristics of this technology are described below.

### 2.1. LoRa

The main innovations introduced by LoRa are low-power consumption and long-range, thus providing a basis for creating a LPWAN (Low Power Wide Area Network) to support the Internet of Things (IoT) and associated smart city applications.

When operating in an unlicensed band, LoRa technology competes with all existing communications in the same band. However, it also allows any entity to install and operate its network without the need for additional licensing, also implying that limitations on the number of messages transmitted are introduced by regulatory domains. In the European case, the limitation is imposed by a duty cycle, which represents the activity rate of the radio channel, with a value of 1% (in most frequencies), indicating that a device cannot transmit for more than 1% in a given period.

LoRa technology is patented and owned by Semtech, Camarillo, California, United States, which is the main manufacturer of the radio components, however, it is supported by the LoRa Alliance organization, composed of several manufacturers and integrators, from where the standardization of the LoRaWAN protocol arose, which allows the creation of a network structure based on LoRa transmission technology.

LoRa technology uses a modulation scheme with spectrum spreading, of the CSS type (Chirp Spread Spectrum), using spreading factors between 7 and 12, depending on the transmission rate and signal level of the connection, spreading factor 12 being the one that introduces better guarantees of the signal reaching the destination, but also the one that has a lower transmission rate, and higher chances of suffering a collision [3]. Additionally, in order to optimize the reception of the signal, an error correction mechanism is used, and the Link Budget of a LoRa transmission can reach values of 168 dB [4]. The Link Budget is the determining factor when considering the network coverage of a device in any environment.

### 2.2. LoRaWAN

The LoRaWAN protocol is a link layer and network layer protocol, including Medium Access Control (MAC) layer protocol, considering the OSI model, allowing sensors with a LoRa radio interface to communicate with applications connected to the Internet. It is promoted by the LoRa Alliance and free to use.

**Figure 1:**
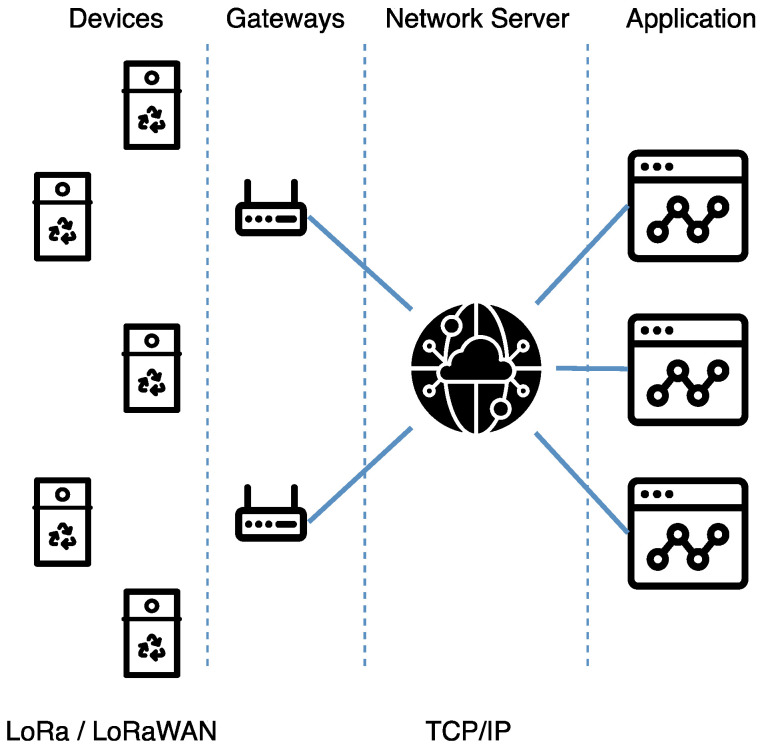
LoRaWAN architecture.

A LoRaWAN network is based on four components, as shown in Figure 1:

1. The sensor device, usually with energy and computational limitations;
2. The gateway, a network element that receives and transmits data from and to devices;
3. The network server, which forwards messages received by a set of gateways to the applications and vice versa;
4. The application, somewhere on the Internet, that receives and sends data to the sensors through the network server.

LoRaWAN defines three device classes. The first, the most used, is Class A, which features devices that are mostly asleep, battery powered, and transmit information (uplink) only when necessary. It is possible to return some information to these devices (downlink) using reception windows that are located after the transmissions from the devices. Class B devices are devices that regularly receive information and as such combine with the network the times when they will wake up to receive that information. Finally, Class C devices are always active and can always receive information when needed.

In terms of security, LoRaWAN specifies two levels of security, the first between the device and the network, the second between the device and the application. This ensures that only the application can interpret the data sent by a device.

**Figure 2:**
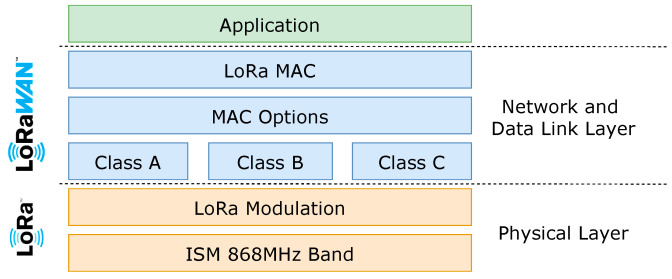
LoRaWAN layers.

The LoRaWAN protocol also introduces extra features, such as the Adaptive Data Rate, which allows the network to negotiate with the devices the parameterization of the physical LoRa transmission, optimizing consumption and spectral efficiency. Figure 2 presents a layer diagram for LoRa and LoRaWAN.

## 3. Related Work

The applicability of LoRa to smart cities has long been studied and researched. In [5] the authors evaluate the applicability of LoRa to smart cities by tracking the number of published works and the same smart city vertical this paper addresses. From here they determined that the waste management is the second most addressed vertical on a smart city by current research, with environment leading the table.

LoRa is not the only technology used for waste management in cities. In [6,7,8,9,10,11,12,13], multiple Low Power Wide Area Network types are used to address the waste management problem, specifying complete solutions from the sensor to the application, addressing intelligent routing of waste trucks [14,15,16] and energy [17,18,19,20,21,22,23,24,25,26,27,28,29,30,31,32,33,34,35,36,37,38,39,40,41,42,43,44,45,46,47,48,49,50] as two of the main issues with this kind of application.

However, regarding the practical application of LoRa based commercial sensors to commercial waste containers, in place and being used daily, the related research is scarce, being [14,51,52] some of the most relevant works addressing the research on the sensor type up to the application and business case, all of them with trials in European cities. In [14], the city of Salamanca, Spain was used as a use case, the authors used sensors developed by themselves together with commercial waste containers (although only surface ones). In [51] the authors also developed the sensors and mostly addressed the business case, using The Things Network as the underlying network for their trials, deployed in Herning, Denmark, the same network as the one used on our own trials. In [52], the authors depict a case study in Luxembourg for improving the waste collection process. The presented case study uses Sigfox as an underlying LPWAN for the filling level sensors placed at the waste containers. In this study, the authors included commercial sensors and waste containers, placing them at indoor scenarios, unfortunately the main objective of this study was the optimization of the waste collection process and not the evaluation of the impact of the different scenarios on the connectivity.

These works mostly use LoRaWAN as a possible network layer and lack trials on the network planning and connectivity requirements when using commercial solutions. This leads us into this work, effectively validating the research on sensors, networks, energy efficiency, and communication technologies when applied to real use cases considering the currently used waste containers in a large city, its landscape, and a network using commercially available equipment.

Another conclusion on the related work is that Europe is leading the research, using LPWAN technologies, to address the smart city vertical of urban waste management, with LoRaWAN being the most prominent underlying network layer.

## 4. Trials

Taking into account the objectives, focused on the application of monitoring solid waste containers, it was essential to carry out a set of trials that would confirm the applicability of the LoRa technology. This applicability must be confirmed at two levels:

- Radio coverage, ensuring LoRa provides the coverage levels required to monitor waste containers installed on the surface and underground;
- Capacity, validating whether the capacity offered by the network for data transmission is sufficient to satisfy the need for the application in question.

Having these objectives in mind, tests were carried out, using gateways and sensors acquired for the purpose, which are presented below.

### 4.1. Network Deployment

In order to support the trials, two gateways were purchased and installed, with different characteristics and placements, in order to increase the validity of the tests. The gateways were integrated into The Things Network (TTN), which provides the backend network component to support LoRa gateways, namely network servers and application servers. Table 1 shows the main characteristics of the gateways used. The gateway installed in the Amoreiras building uses the existing firefighter communications tower as support for the purpose.

**Figure 3:**
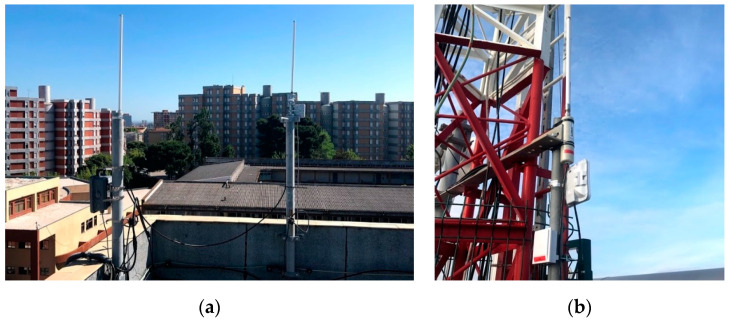
LoRa gateways deployed at (**a**) ISEL and (**b**) Amoreiras.

Figure 3 shows the installation of these devices. It should be noted that the equipment installed at ISEL is operating in diversity mode, with two antennas, in order to increase the probability of success in receiving messages.

### 4.2. Sensors

Considering the aim and purpose of the tests, it is intended that they are based on sensors with different characteristics, allowing meaningful conclusions. For this purpose, two types of filling level monitoring sensors for waste containers were purchased, both commercial, but from different manufacturers and different cost ranges:

- IoTsens Waste Sensor, high-end sensor, with quality specifications and higher costs (€300);
- Dingtek DF702, sensor with lower specifications, also corresponding to lower costs (€70).

Both sensors use the general principle of ultrasound measurement, in which the sensor is placed on top of the container and measures the distance between the sensor and the waste. Thus, a lower distance entails a higher filling level. This form of measurement has as its main advantages, the ease of use and low cost and as disadvantages, its inability to deal with situations of nonhomogeneous filling levels and false positives in situations of low-density waste (such as cardboard). This implies that the position of the sensor placement and its processing capacity greatly influence the reported level.

#### 4.2.1. IoTsens Waste Sensor

**Figure 4:**
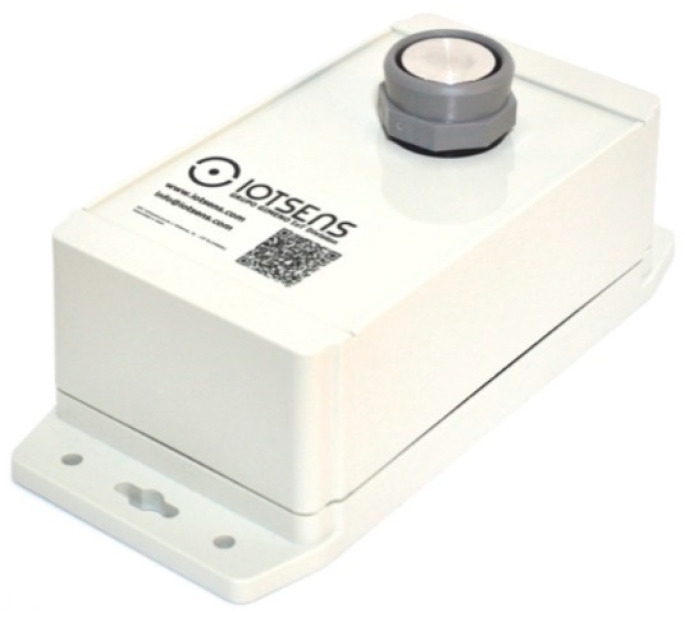
IoTsens waste sensor.

In Figure 4, the IoTsens sensor is shown, in which the main highlight of this device is the volumetric sensor used, manufactured by Maxbotix, Brainerd, Minnesota, United States, allowing to measure different distances up to 5 m. Additionally, noteworthy is its ability to measure temperatures (allowing the detection of fires), inclination of the container (for detecting waste collection routines) and battery capacity (26 Ah).

In terms of communications, IoTsens uses a modem from RisingHF, with a PCB (Printed Circuit Board) printed antenna.

#### 4.2.2. Dingtek DF702

**Figure 5:**
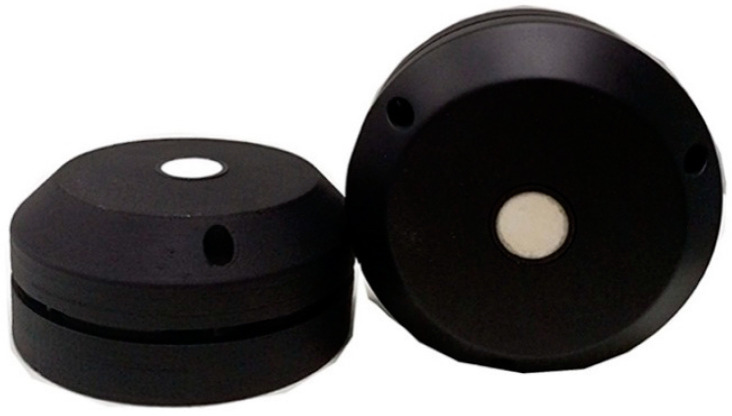
Dingtek DF702.

The Dingtek DF702 device, shown in Figure 5, is a sensor with a lower cost, associated with its lower specifications, namely the limitation in measuring distances greater than 2 m and a battery of only 7 Ah. These limitations prevent its installation in some scenarios, namely in deep underground containers.

Like the IoTsens sensor, the modem used is RisingHF, however, using a helical antenna.

Despite the low cost, the Dingtek DF702 sensor is more robust, observable by the thickness of the box and in the form of a seal to guarantee its tightness.

#### 4.2.3. Sensor Deployment

Given the characteristics of the sensors, we opted to apply IoTsens to underground waste containers and Dingtek to Iglô and Cyclea. For this purpose, metal supports compatible with the containers and sensors under test were designed and manufactured. Using metal as a support introduces side effects to the transmission. These side effects were considered but during the waste collection process the containers are subject to a number of external forces, from impacts during the pick-up, imposing that the support should be very robust, hence the only practical option was to use a metallic support.

**Figure 6:**
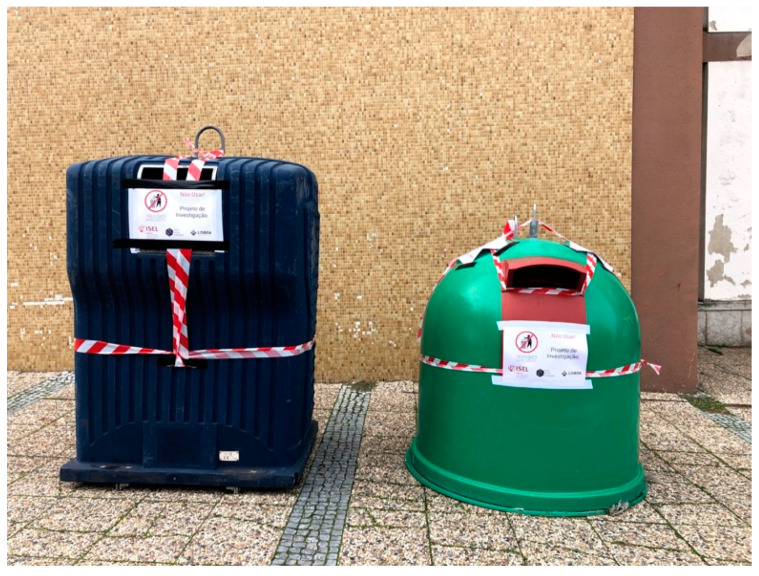
Cyclea (blue) and Iglô (green) containers installed at ISEL.

Figure 6 shows both containers (Iglô and Cyclea) at ISEL during its installation phase (later, containers were deployed to their usual locations). Dingtek DF702 sensors were installed in both containers, using different fixings, 3D custom designed, and adapted to each type of container.

**Figure 7:**
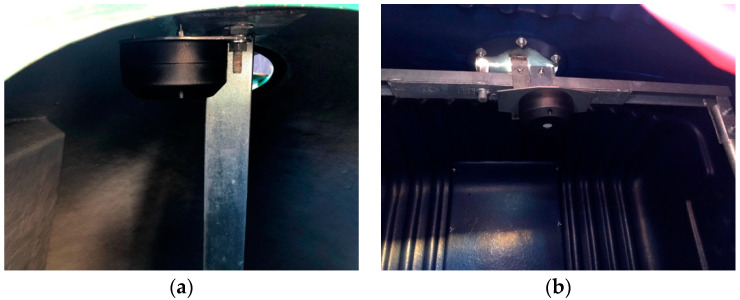
Dingtek DF702 installed in the containers: (**a**) installed in an Iglô; (**b**) installed in the Cyclea.

In Figure 7 it is possible to observe the installation of the sensor in an Iglô and in a Cyclea container. Cyclea containers are used for multiple types of recyclable waste, however, they share the same sensor.

**Figure 8:**
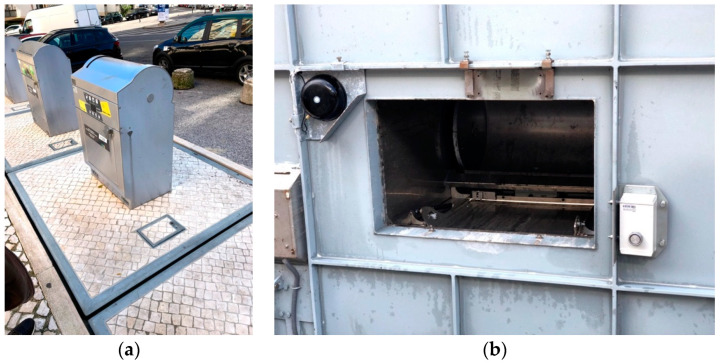
IoTsens installed on the underground waste bin: (**a**) general view; (**b**) cover underside view.

Figure 8 shows the installation of the IoTsens Waste Sensor in an underground container. The installation was made using two holes already existing on the underside the metallic cover. In this same cover, it is possible to observe the legacy sensor installed and in use, this sensor needs an external cellular antenna to communicate correctly. The IoTsens sensor was installed in a position that facilitates the measurement, but that technically presents greater difficulties in the transmission of information, due to the distance from the waste collection shaft. This position was chosen for reusing existing wholes, not requiring any new drilling, but also for introducing more challenges to the correct operation.

### 4.3. Use Cases

During the tests, three different use cases were selected. The first at a short distance (≈100 m), the second at an average distance (≈1 km) and a third at a longer distance (≈5 km). These three use cases allowed to evaluate the capacities of the sensors to transmit information in different conditions of radio propagation. The short distance scenario would have an expected success and served as a baseline for the others.

**Figure 9:**
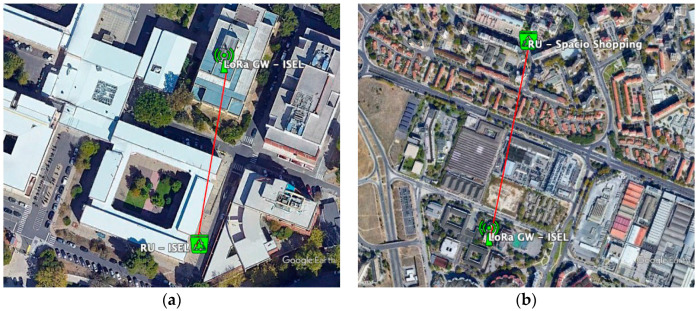
Map showing the distances from the LoRa GW at ISEL to sensors at (**a**) the underground containers (RU); (**b**) at ISEL.

**Figure 10:**
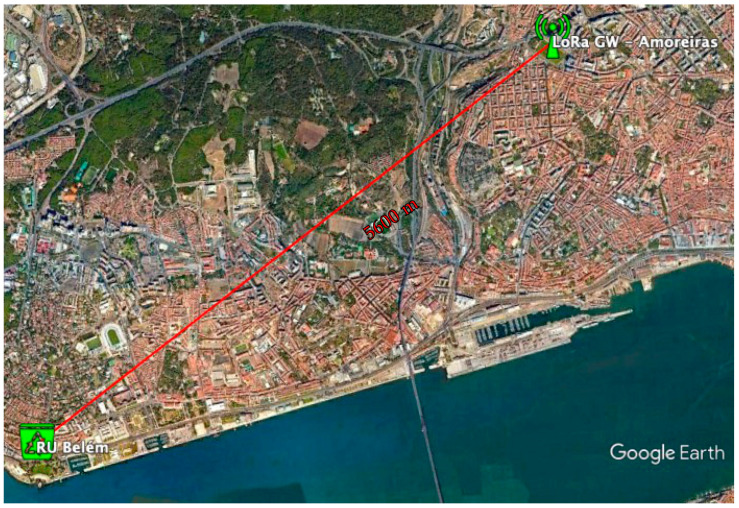
Map showing the distance from the sensor to the LoRa Gateway at Amoreiras.

For the short distance use case, a sensor in an Iglô was installed in ISEL (N38°45′19.7″ W9°06′58.0″), visible in Figure 9, the medium distance sensor was installed in an underground container, next to Spacio Shopping (38°45′42.3″ N 9°06′52.3″ W) and finally the Cyclea container was installed at a long distance, at Largo da Princesa in Belém (38°41′41.9″ N 9°12′57.4″ W), visible in Figure 10.

## 5. Evaluation

The test results, carried out according to the conditions defined in the previous section, assess the applicability of the LoRa technology to the application of measurement and level of filling of urban waste containers. Thus, the following section presents the radio coverage results obtained in the different scenarios.

Results will also be presented regarding the impact of installation conditions on the level of radio coverage, which may be useful, not only within the scope of this study, but also in the context of producing tender specifications, allowing correct radio coverage requirements to be established for different applications.

### 5.1. Radio Coverage

As expected, the reduced distance scenario presented no challenges to the LoRa technology, with all data received. However, the longer distance scenario (≈ 5 km), proved to be too demanding, and not a single transmission of the sensor installed in Belém was ever heard at Amoreiras.

**Figure 11:**
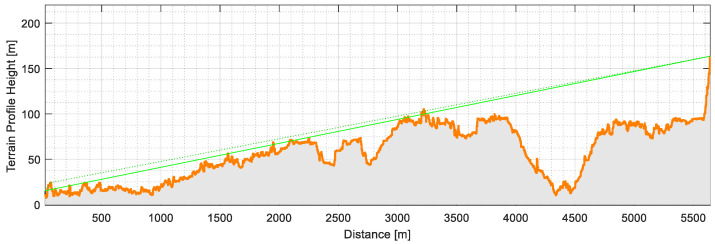
Distance profile between the waste container at Belém and the LoRa gateway at Amoreiras.

A more detailed analysis of the terrain profile and the longer distance, shown in Figure 11, shows that there will be no line of sight between the location of the container installation and the nearest gateway, due to the morphology of the terrain, namely Monsanto (a large municipality managed forest/park), but also due to the urban landscape existing on the radio link.

The scenario of the underground container presented challenges in terms of good reception of the signal due not only to the installation site, but also due to the metallic construction of the container, particularly the steel cover and pavement applied on the cover upper surface. This installation context prevents, for example, direct radio communication with the legacy sensors currently installed with GSM/GPRS technology, requiring the placement of an external antenna, outside of the waste container, in order for it to work correctly. Installing an external antenna voids the watertightness of the waste container and is undesirable.

**Figure 12:**
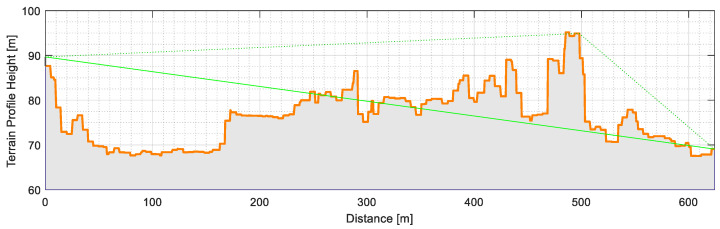
Distance profile between the waste container at Spacio Shopping and the LoRa gateway at ISEL.

Figure 12 shows the terrain profile between the ISEL gateway and the underground waste container, where it is also visible that there is no line-of-sight communication. This factor aggravates the existing bad radio conditions, but it is a good opportunity to study the performance of technology in unfavorable situations. The figure also shows the direct beam and diffraction path. This high obstruction results from buildings on the radio propagation path to the nearer gateway.

**Figure 13:**
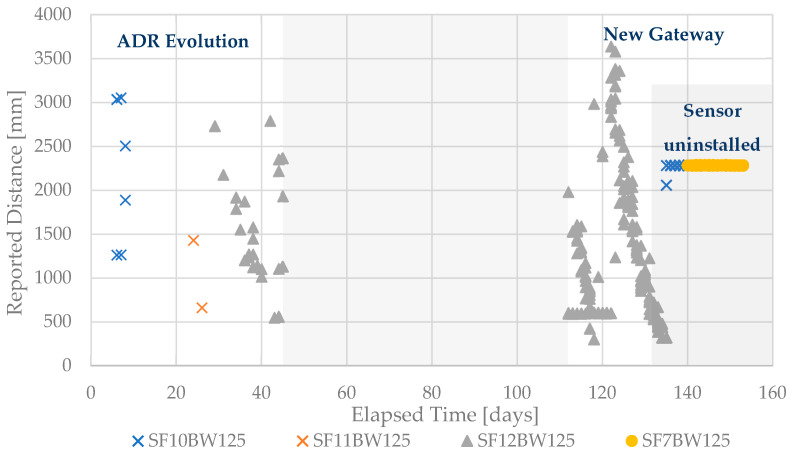
Measurements received from the underground container at Spacio Shoping.

In Figure 13, the values reported by the filling level measurement sensor are presented from its installation to its removal. It should be noted that for about two months, starting in day n + 45, up to day n + 112, there were no measurements, due to a fault in the existing gateway. This situation led to the need to replace the gateway with new equipment that entered service on day n + 112.

The evolution of the spreading factor due to the ADR (Adaptive Data Rate) of the LoRa is visible in the initial part of the chart. This automatic adaptation mechanism allows the system to switch spreading factor (between 7 and 12) according to the radio conditions, allowing to optimize the relationship between the transmission rate, duty cycle and energy consumption, leading to greater spectral and energy consumption efficiency. A lower spreading factor led to a higher transmission rate and a lower channel occupation time. However, it requires a better signal-to-noise ratio (SNR), which is only possible if the attenuation of the radio link is low and the level of noise and interference too. On the contrary, a maximum spreading factor leads to a lower rhythm and consequently much longer emission times, with greater energy consumption. Thus, the system uses the spreading factor that optimizes this equation.

In the figure it is possible to observe that the first messages were received with SF10BW125, which corresponds to a spreading factor of 10. However, there is a lack of continuous reception of messages, caused by a high error rate. Thus, it is possible to observe the transition to the spreading factor 11 and, later, to the maximum of 12. From that point, the frequency of receiving messages increases. Finally, after collecting the sensor, for a more favorable position (inside an office at ISEL), the spreading factor decreased again, until the minimum value of 7 (yellow circles in the figure).

It should be noted that the validation of the reported distance to waste values is not the object of this study. However, it is possible to observe that, after the new gateway goes into operation, the gradual filling pattern of the container is clearly identified and a situation in which the distance returns to the maximum value, which should correspond to the collection of accumulated waste, thus leading the sensor to report maximum distance from the sensor position to the bottom of the container.

### 5.2. Deployment Conditions Impact

**Figure 14:**
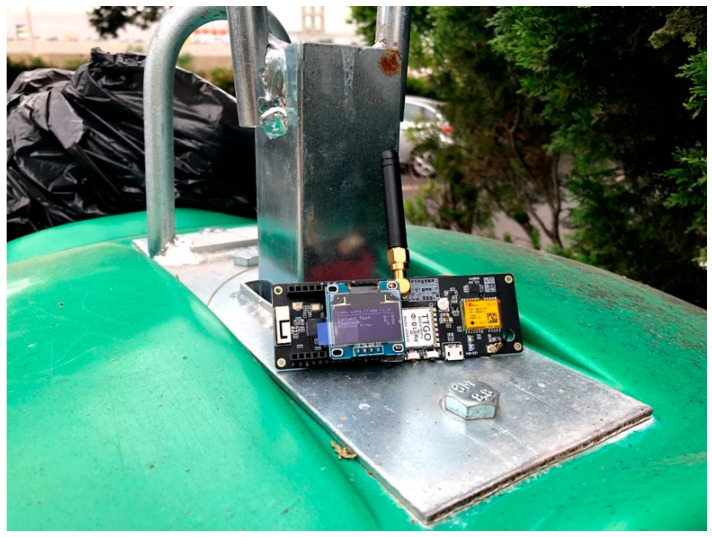
LoRa probe installed on top of an Iglô.

In order to deepen the knowledge about the limitations imposed by the material used in the containers, tests were carried out that allowed to determine the impact of different materials on the transmission. These tests were then carried out for Iglôs (Empty and Full) and for underground containers (Full). For the tests, a probe was developed, visible in Figure 14, which sends five messages of two different dimensions (4 and 8 bytes), using each of the different parameters of the LoRa modulation (SF7 to SF12), which totals 60 messages sent in each of the tests. The sizes depict the actual size of the data sent by the Dingtek (4 Bytes) and IoTsens (8 Bytes) sensors. Algorithm 1 depicts the implementation of the network assessment procedure.
**Algorithm 1 Network Probe****Input:** Datarates and Payloads to be tested**Output:** Perceived RSSI (Received signal strength indication) of each message1:  initialize radio at device2:  initialize application connected to TTN3:  **for each** payload **in** payloads[] **do**4:     **for each** datarate **in** datarates[] **do**5:       set datarate6:       send payload7:       sleep in order to comply with the duty cycle8:  collect RSSI values from application

**Figure 15:**
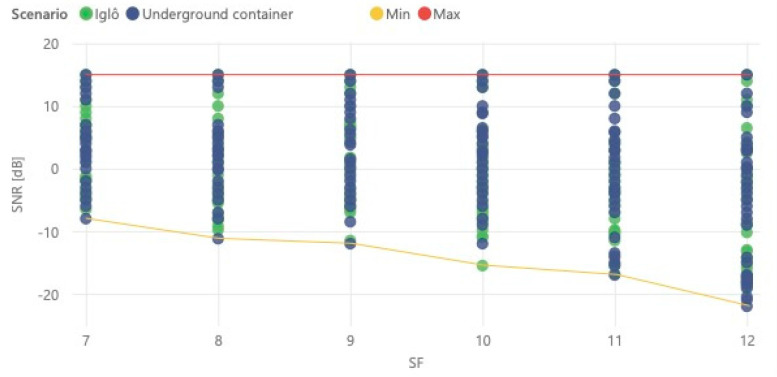
Received message SNR versus spreading factor.

Since the ADR mechanism was disabled during the tests, and the spreading factor is changed cyclically, it is difficult to infer the relationship between the message SNR (Signal-to-Noise Ratio) and the SF used for transmission. However, it is possible to observe in Figure 15 the minimum SNR values for each SF, taking into account the limitations of LoRa modulation. In the figure, it is possible to observe, for each spreading factor, the measured SNR of each message. Thus, in addition to the minimum value, which varies between −9 and −22 dB, we can verify that the maximum reported value is 15 dB.

**Figure 16:**
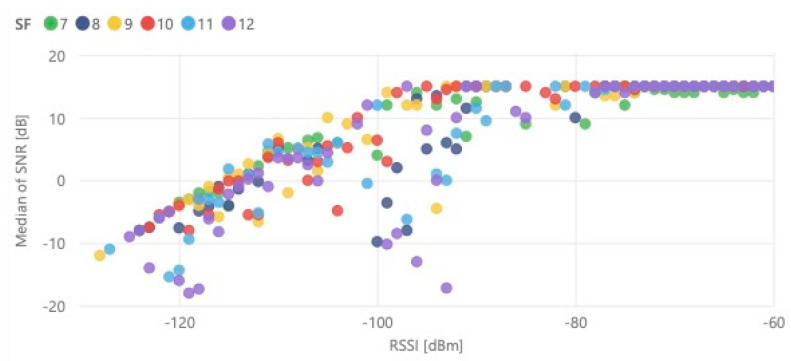
Received message SNR versus RSSI.

Measuring received message SNR and RSSI, we can check the dependency of these two radio performance indicators. The scatter plot presented in Figure 16 confirms the linearity of RSSI and SNR ratio between −130 and −100 dBm, where the maximum value and SNR is proportional to RSSI. For RSSI higher than −100 dBm, the SNR will lead to saturation on 15 dB. It is also possible to observe the influence of interfering signals in the communication, causing high dispersion of SNR in some cases. This situation is justified because LoRaWAN uses the ISM (Industrial, Scientific and Medical) band of 868 MHz, so, depending on the use case, the radio channel potentially will present a high occupancy, and consequently, higher interference level.

#### 5.2.1. Iglô

**Figure 17:**
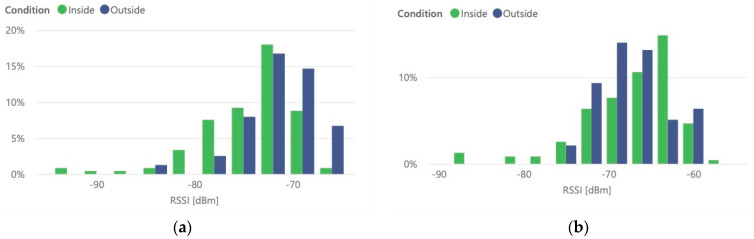
Iglô container received message signal strength indication: (**a**) with the container full and (**b**) with the container empty.

Four tests were performed on different glass containers, reflecting the combinations of the state of the container, full and completely empty, and test conditions, inside and outside. Table 2 shows an analysis on the signal level (RSSI—Received Signal Strength Indicator) of the messages sent by the probe when installed inside and outside each one. The values result from the tests, where measured RSSI values distribution is presented in Figure 17. We can observe the difference on received signal strength indication samples distribution, where inside measurements present a higher statistical variance than when the probe was outside. This behavior is consistent on both container states.

In terms of impact on the signal, it is observable that the Iglô introduces an average attenuation of 6 dB when empty and about 2 dB when full. It should be noted that the measurements were made in containers whose model is identical but were in different positions within ISEL. The measurements were also made on different days and at different times of the day, so the comparative analysis should focus on inside/outside conditions, that is, the difference in the RSSI value, representing the impact of the waste container build material.

#### 5.2.2. Underground Container

**Figure 18:**
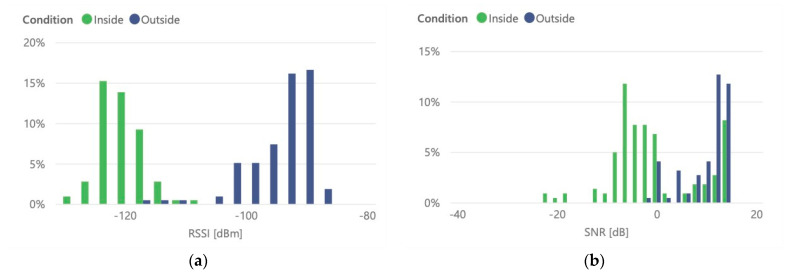
Underground container received messages: (**a**) radio quality RSSI; (**b**) SNR measurements.

To test the underground container radio coverage and to obtain penetration attenuation, tests were performed positioning the LoRaWAN transceiver inside and outside of the container. Figure 18 presents the probability distribution of RSSI and SNR values of received message, for each condition. It is possible to observe the influence of container on received signal level and quality.

The underground container, as expected by its steel construction, introduces a much greater attenuation, reaching average attenuation values above 26 dB. These values are visible in Table 3. This fact will imply that in terms of dimensioning the radio network it will be necessary to account for this increase in terms of link budget, in order to allow the use of sensors without external antennas.

## 6. Conclusions

The objective of this study was to verify the applicability of LoRa technology to support the transmission of the filling level of waste containers throughout the city of Lisbon. A set of tests were carried out, under different conditions and scenarios, allowing the conclusions presented here to be supported by empirical knowledge, which complements the existing technical and scientific information.

In the case of coverage over long distances, the failure to receive messages confirmed the criticality of rigorous network planning, in order to ensure adequate radio coverage of the city. It is therefore essential to adopt link budget margins, compatible with existing conditions, during network planning. It also proved the importance of the characteristics of the antenna used in the sensor and its impact on the performance of radio communication.

Regarding the coverage of underground waste containers, a situation that was expected to be more unfavorable than the other different verticals, radio coverage that allowed for communication was possible, even in a no line of sight situation. Thus, the tolerance to high levels of attenuation of LoRa was confirmed. It was therefore interesting to verify the performance of this technology, in comparison with the solution currently installed (GSM/GPRS), under the same installation scenario, even in the most adverse conditions.

Based on the results presented, it is possible to conclude that LoRa allows to fully support the transmission of data to monitor the filling level of waste containers. This possibility applies at the following levels:

- Capacity, where it was possible to confirm that the capacity offered by technology for data transmission is sufficient to satisfy the need for the application in question, even in unfavorable conditions, which imply a lower transmission rate;
- Radio coverage, showing the feasibility of using LoRa technology to support communication with sensors installed inside the different types of containers. This possibility was validated in the most unfavorable situation, namely in underground containers, with additional attenuations, associated with the penetration of the radio signal.

Ultimately, this research led to the deployment of a municipality wide local area network to support the Waste Management Department and the Public Park Management Department of the Lisbon Municipality as well as preparing the grounds for other initiatives promoted by Lisbon City Council.

The current ecosystem surrounding smart cities sets pace to the rise of technologies such as LoRa, that proves to be flexible and fitted to the most demanding smart city verticals such as urban smart waste management. Managing an increasing number of waste types with a common fleet of waste collection trucks proves to be difficult. With this restriction in mind, the continuous monitoring of the waste level of containers, helps to ensure a better management of the waste collection truck fleet, by only collecting waste from containers that are full, effectively improving the overall fleet efficiency. An improved efficiency leads to the increase of the number of recyclable types of waste containers, which will indirectly lead to less landfill usage of unrecyclable waste. The trend on the landfill usage is that it is decreasing for the past years at European municipalities [53]. The results presented in this paper allow a municipality to support the decision process on the selection of an LPWAN for the second most addressed smart city vertical. This is probably one of the most demanding considering LPWAN connectivity, given the high number of devices and their installation characteristics, particularly at underground waste containers.

## Figures and Tables

**Table 1:** Gateways used.

Location	Model	Antennas	GNSS Position	Altitude
ISEL	Cisco IXM-LPWA-800-16-K9	2	N38°45′22.7″ W9°06′57.3″	100 m
Amoreiras	Lorix One	1	N38°43′24.0″ W9°09′47.5″	175 m

**Table 2:** RSSI measurements on Iglôs containing glass.

Scenario	RSSI Value (dBm)				Probe Message Count
	Min	Max	Avg	StdDev	
Iglô full					
Inside	−91	−66	−73.75	5.18	60
Outside	−83	−65	−71.82	3.69	60
Difference	−8	−1	−1.93		
Iglô empty					
Inside	−85	−62	−70.07	5.18	59
Outside	−72	−59	−63.98	3.38	59
Difference	−13	−3	−6.09		

**Table 3:** RSSI measurements on underground containers.

Scenario	RSSI Value (dBm)				Probe Message Count
	Min	Max	Avg	StdDev	
Underground Container					
Inside	−127	−111	−121.31	2.32	49
Outside	−115	−87	−94.90	6.29	59
Difference	−12	−24	−26.41		

## Data Availability

The data presented in this study are available on request from the corresponding author. Data requests are managed on a per request basis due to requirements of the contract signed between CML and ISEL.
